# Transitions through the HIV continuum of care in people enrolling in care with advanced HIV disease in Latin America

**DOI:** 10.1016/j.ijregi.2024.100550

**Published:** 2024-12-18

**Authors:** Pablo F. Belaunzarán-Zamudio, Peter F. Rebeiro, Yanink Caro-Vega, Jessica Castilho, Brenda E. Crabtree-Ramírez, Carina Cesar, Claudia P. Cortes, Fernando Mejía, Marco Tulio Luque, Vanessa Rouzier, Guilherme Calvet, Catherine C. McGowan, Juan Sierra - Madero

**Affiliations:** 1 Instituto Nacional de Ciencias Médicas y Nutrición Salvador Zubirán, Mexico City, Mexico; 2 Vanderbilt University School of Medicine, Nashville, USA; 3 Fundación Huesped, Buenos Aires, Argentina; 4 Facultad de Medicina Universidad de Chile and Fundación Arriarán, Santiago, Chile; 5 Universidad Cayetano Heredia, Lima, Peru; 6 Instituto Hondureño de Seguridad Social, Tegucigalpa, Honduras; 7 Le Groupe Haïtien d'Etude du Sarcome de Kaposi et des Infections Opportunistes in Port-au-Prince (GHESKIO), Port-au-Prince, Haiti; 8 The Oswaldo Cruz Foundation (Fiocruz), Instituto Nacional de Infectologia Evandro Chagas (INI), Rio de Janeiro, Brazil

**Keywords:** HIV, AIDS, Continuum of care, Latin America, Advanced HIV disease

## Abstract

**Objectives:**

Advanced HIV disease (AHD) at HIV care enrollment is common in Latin America and may bias cross-sectional care continuum estimates. We therefore explored the impact of AHD on HIV care continuum outcomes using a longitudinal approach.

**Methods:**

We analyzed trajectories of 26,174 adult people with HIV enrolled at Caribbean, Central and South America network for HIV epidemiology (CCASAnet) sites (2003-2019) using multi-state Cox regression across five stages: (i) enrolled without antiretroviral therapy (no-ART); (ii) on ART without viral suppression (viral load ≥200 copies/m; ART + non-VS); (iii) on ART with viral suppression (viral load <200 copies/ml; ART + VS); (iv) lost to follow-up; (v) death. We defined AHD as clusters of differentiation 4+ count <200 cells/µl and/or an AIDS-defining illness at enrollment.

**Results:**

People with HIV with AHD had a shorter time with no-ART, a similar time ART + non-VS, but less time ART + VS before 2013 than non-AHD. After 2013, time with no-ART decreased but the 5-year probability of transitioning from no-ART to ART + VS decreased in both groups. The time spent virally suppressed while in care was low, overall. Risk of loss to follow-up and death was persistently worse among adults with AHD.

**Conclusions:**

Using a longitudinal approach to assess the HIV continuum of care provided insight into limitations in HIV care provision in our region previously underexplained by cross-sectional assessments.

## Highlights

• We assessed the impact of advanced HIV disease in the HIV care continuum using longitudinal analyses.
• Overall the time spent virally suppressed while in care was low.
• A longitudinal approach to assess the continuum allows insights into HIV care limitations.
• Our approach provided evidence that high advanced HIV disease biases cross-sectional estimates.
• Improving retention in care and antiretroviral therapy adherence are needed to maximize antiretroviral therapy as prevention benefits.

## Introduction

Late diagnosis and advanced HIV disease at linkage-to-care and antiretroviral therapy (ART) initiation remain common in Latin America [[1], [2], [3]]. In previous studies within the Caribbean, Central and South America network for HIV epidemiology (CCASAnet), we documented the negative impact of presentation to care with advanced HIV disease and late ART initiation on different clinical outcomes and clinical care [[1], [2], [3]]. People with HIV (PWH) starting ART with advanced disease have higher mortality, higher rates of early ART changes, higher rates of loss-to-follow-up (LTFU) after ART initiation, and a higher risk of needing third-line ART regimens [[1], [2], [3], [4]]. The majority of deaths among PWH enrolled in care at clinical sites in CCASAnet can be attributed to late presentation [1].

The HIV care continuum is a powerful framework for monitoring major outcomes of HIV programs [5,6] and assessing targets necessary to control the epidemic [7]. However, outcome definitions, stages evaluated, and populations monitored through care continuum studies are heterogeneous and themselves topics of interest [8,9]. Two methodologic approaches have been used: cross-sectional and longitudinal. While cross-sectional analyses are common, they may lead to biased conclusions [8]. Longitudinal approaches allow researchers to estimate the flow of people through stages of the continuum and the time spent in each stage [8,10]. Further, a longitudinal framework makes more complete use of continuous, interrelated, and temporally varying data types [8].

In 2022, UNAIDS estimated 72% (95% confidence interval [CI]: 64-80) of PWH in Latin America were receiving ART, and 66% (95% CI: 59-74) were virally suppressed. Since 2010, an increase of 8% in new HIV infections has been reported in Latin America, and the percentage of PWH presenting for care with advanced HIV disease (AHD: defined as PWH newly diagnosed and linked to care with clusters of differentiation [CD4+] lymphocyte counts <200 cells/µl) has remained ≥25% [11]. In CCASAnet, we recently assessed the HIV care continuum and identified significant improvements in retention in care, ART use, and viral suppression over time, but disparities for key populations remained [12]. Assessing the impact of late diagnosis and AHD on specific stages of the care continuum can provide further guidance to expand access to HIV testing, improve linkage-to-care in our region, and provide insight into later stages.

In this analysis, we examined the impact that enrolling in care with advanced HIV disease has on achieving care continuum benchmarks longitudinally. We are focusing on advanced HIV disease (AIDS or CD4<200 cells/µl) at enrollment rather than in late presentation (AIDS or CD4 <350 cells/µl [13]) because this is the most common form of late presentation in our region: the median CD4 count at enrollment in CCASAnet centers is 198 cells/µl (p25-p75, 68–81) and people with advanced disease account for 75% of all patients with late presentation, which comprises 74% of people in our cohort. While people with AHD are more likely to start ART, we hypothesize that they are less likely to be retained in care and achieve HIV viral suppression in both the short and long term. We quantified the effect of AHD across multiple stages of the care continuum, accounting for transitions and the time-varying nature of care continuum outcomes using a longitudinal multi-state model.

## Methods

### Study population

We included ART-naïve PWH ≥18 years old who enrolled in care from January 1st, 2003, until March 10th, 2019, in clinical sites of CCASAnet. CCASAnet is a multinational cohort collaboration of HIV clinical centers in seven Latin American countries: Centro Médico Huésped, Buenos Aires, Argentina (CMH-Argentina); Instituto Nacional de Infectologia Evandro Chagas, Fundação Oswaldo Cruz, Rio de Janeiro, Brazil (FC- Brazil); Fundación Arriarán, Santiago, Chile (FA-Chile); Le Groupe Haïtien d'Etude du Sarcome de Kaposi et des Infections Opportunistes, Port-au-Prince, Haiti (GHESKIO-Haiti); Instituto Hondureño de Seguridad Social and Hospital Escuela, Tegucigalpa, Honduras (IHSS/HE-Honduras); Instituto Nacional de Ciencias Médicas y Nutrición Salvador Zubirán, Mexico City, México (INCMNSZ-Mexico); and Instituto de Medicina Tropical Alexander von Humboldt, Lima, Perú (IMTAvH-Peru) [14]. We included data from all sites in the primary analysis. We conducted a sensitivity analysis excluding Haiti due to the lack of available viral load (VL) data at the site over the entire observation period. We excluded people without CD4+ lymphocyte count (CD4) measures or AIDS-defining event (ADE) status at enrollment. Individuals contributed data from clinic enrollment until their last clinic visit or study closure (Table 1).

**Table 1:** Summary of patient demographics and outcomes by calendar group and late presentation status at enrollment.

	Enrolled before January 1, 2013 N = 14,497		Enrolled after January 1, 2013 N = 11,677	
	With late presentation (n = 8821, 61%)	Without late presentation (n = 5676, 38%)	With late presentation (n = 5936, 51%)	Without late presentation (n = 5741, 49%)
Age in years	36.7 (30-44.5)	33.5 (27-42)	35.4 (28.6-44.2)	29.9 (24.8-38)
Male sex at birth	5700 (65%)	3257 (57%)	4011 (67%)	4090 (71%)
School level				
Less than Primary	1330 (15%)	743 (13%)	902 (15%)	570 (10%)
Secondary	4542 (51%)	2729 (48%)	3211 (54%)	2747 (48%)
University/Post-Graduation	1237 (14%)	1015 (18%)	1214 (20%)	1986 (34%)
Unknown	1712 (19%)	1189 (21%)	609 (10%)	438 (7.6%)
CD4+ at enrollment, (cells/µl) Missing CD4+	86 (36-161) 699 (7.9%)	357 (273-495) -	98 (41-174) 1335 (22%)	410 (304-563) -
History of AIDS-defining event at enrollment	5222 (59%)	-	3590 (60%)	-
HIV risk acquisition				
Heterosexual contact	2593 (29%)	1232 (22%)	3022 (51%)	1859 (32%)
Men who have sex with men contact	1863 (21%)	1621 (29%)	1719 (29%)	2899 (50%)
Other	61 (<1%)	30 (<0.5%)	45 (<1%)	24 (<0.5%)
Unknown	4304 (48%)	2793 (49%)	1150 (19%)	959 (17%)
Follow-up time (months)	92.80 (25.9-134)	94.03 (47.8-128)	22.8 (9.6-44.2)	24.5 (10.3-45.9)
Year of enrollment	2008 (2006-2010)	2009 (2007-2011)	2016 (2014-2018)	2016 (2014-2017)
Started ART in follow-up	8393 (95%)	5279 (93%)	5552 (93%)	5075 (88%)
Deaths	1660 (19%)	382 (6.7%)	641 (11%)	124 (2%)
Lost to follow-up	4195 (47.5%)	3210 (56.5%)	2320 (39.1%)	2827 (49.2%)
Site				
Argentina	143 (1.6%)	224 (3.9%)	43 (<1%)	138 (2.4%)
Brazil	1065 (12%)	955 (17%)	573 (9.6%)	631 (10.9%)
Chile	643 (7%)	776 (13.7%)	728 (12%)	1384 (24%)
Haiti	3844 (43%)	2614 (46%)	2623 (44%)	1,619 (28%)
Honduras	689 (7.8%)	112 (1.9%)	125 (2.1%)	86 (1.5%)
Mexico	595 (6.7%)	257 (4.5%)	278 (4.6%)	287 (4.9%)
Peru	1842 (21%)	738 (13%)	1566 (26.3%)	1596 (28%)

CD, clusters of differentiation.

Note: Categorical frequencies are presented as n(%) and continuous variables as median(interquartile range). Maximum last date by site: In Argentina: 2021–03–22; Brazil: 2020–08–20; Chile: 2021–04–28; Haiti: 2020–12–30; Honduras: 2020–08–31; Mexico: 2021–01–26; Peru: 2019–12–31. Number of deaths and lost-to-follow-up patients at the end of follow-up.

### Exposure, outcomes, and definitions

The exposure of interest was advanced HIV disease presentation at cohort enrollment (AHD). We defined AHD as enrollment in care with a CD4 <200 cells/µl or a history of any ADE at enrollment (defined using 2008 CDC criteria [15]). We defined AHD using the closest CD4 measurement to the enrollment date within 360 days before and up to 90 days after. Our outcomes of interest were the mean times spent during a single period in various stages of the care continuum (described in detail below) and the probability of transitioning from one stage to another. We also report the estimated total time spent in each stage, which is the sum of time over all periods a participant spent in that particular stage [16].

### Specification of a multi-state model

**Figure 1:**
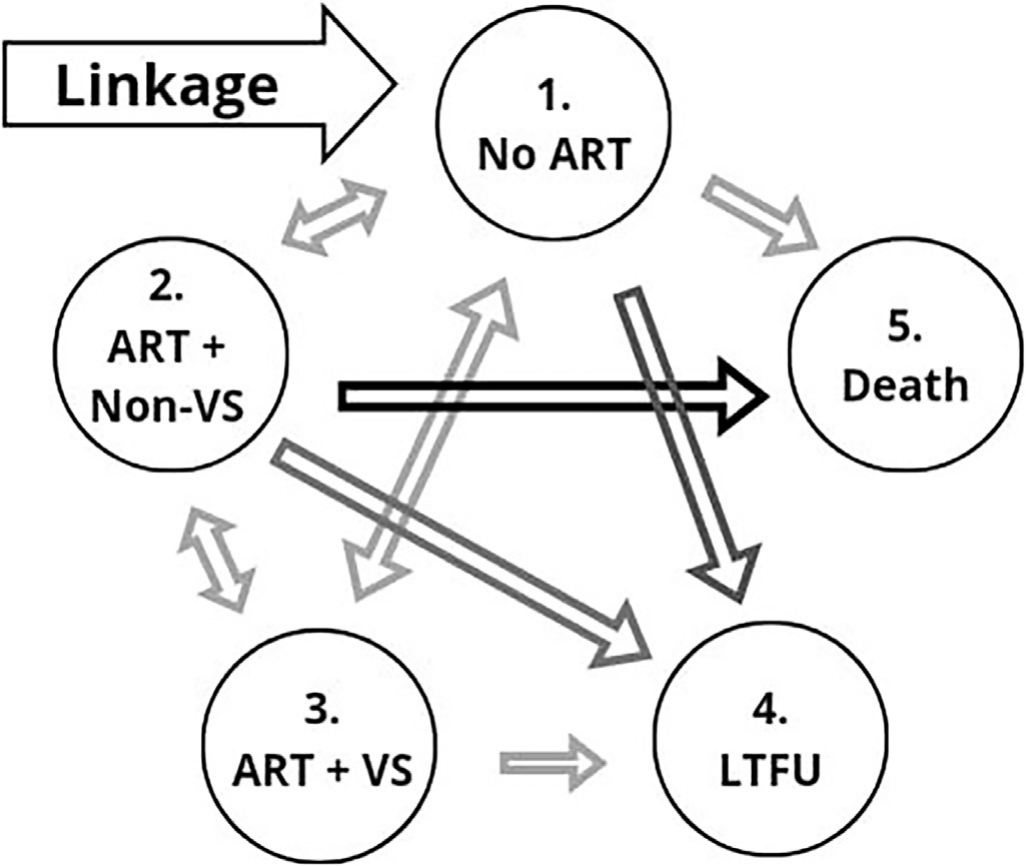
Multi-state model. Note: The states of the model, also stages in the care continuum, are: 1. No-ART: enrolled in care not receiving antiretroviral therapy; 2. ART + non-VS: on ART without viral suppression (HIV-1 RNA viral load ≥200 copies/ml) or not having an HIV-RNA measurement for more than a year; 3. ART + VS: on ART with viral suppression (viral load <200 copies/ml); 4. LTFU: lost to follow-up (≥1 year between last visit and cohort closure date); or 5. all-cause death. States 4 and 5 are considered absorbing states.

We employed a multi-state model to examine the care continuum, describing the flow of individuals in continuous time through discrete and finite states (here, stages of the care continuum), quantified by transition intensities, which depend on time and other explanatory variables [17]. We modeled the movement of PWH between five care continuum stages in continuous time: (i) no-ART: in care but not receiving ART; (ii) ART + non-VS: on ART without viral suppression (HIV-1 RNA VL ≥200 copies/ml) or on ART but not having a VL for more than a year); (iii) ART + VS: on ART with viral suppression (VL <200 copies/ml); (iv) LTFU (≥1 year between last visit and cohort closure date); or (v) All-cause death (Figure 1). We considered only lost-to-follow-up and death as absorbing states (i.e., patients were unable to transition to another state after entering either of these two states). In our model, being in care is implicitly defined as being in any state of the continuum except for LTFU or death. Additional details are shown in the Supplementary Material.

### Statistical analysis

We described characteristics at enrollment among the whole cohort and stratified by AHD vs non-AHD groups: age, sex assigned at birth, probable HIV acquisition risk (men who have sex with men, heterosexual contact, persons who inject drugs, other/unknown), CD4 (cells/µl), ADEs, education level, clinical site, and calendar year of enrollment. We estimated the mean time spent in each stage of the continuum of care comparing AHD and non-AHD groups using the *msm* package in R [16]. We used the estimated total time in follow-up to calculate the percentage of time that a patient spent in each specific stage by AHD status. We stratified this analysis into two groups: those enrolled before 2013 and those enrolled after. We selected 2013 to align with the World Health Organization (WHO)’s expanded eligibility criteria for ART initiation among people with CD4 <500 cells/µl, giving priority to those with advanced HIV disease (WHO stages 3 and 4 or CD4 <350 cells/µl) [18]. This year was also chosen given previous CCASAnet investigations showing that the time to ART initiation decreased significantly after 2013 in our cohort, with no further improvements after 2015 (the start of the “treat-all era” per WHO recommendations to initiate ART among all PWH regardless of CD4) [2].

We used a multi-state multivariable Cox regression model including AHD status, age category (<35, 35-50, ≥50 years), sex assigned at birth, and HIV acquisition risk to estimate mean time spent in each stage, probabilities of transitions to different stages at 1- and 5-years post-enrollment, and adjusted hazard ratios (aHR) and their 95% CIs for transitioning between states. We repeated all analyses in a sensitivity analysis excluding Haiti to reduce the influence of deterministically missing VL data on inferences*.* We also conducted several additional sensitivity analyses addressing changes in the definitions of our primary exposure and of states comprising the care continuum. In the first, we included a new stage to incorporate prospective losses to follow-up. This allowed us to assess the contribution of participants who returned to care after being disengaged from clinical care for a year or more, which starts 365 days after one registered visit and ends on the date of the next consecutive registered visit for any reason or death, using AHD as the exposure. In the second, we used late presentation (AIDS or CD4 <350cells/µl), instead of AHD as the exposure. Finally, in the third, we included the site as a covariate in the original multivariate model. We report detailed results for all sensitivity analyses in the supplementary materials.

## Results

### Characteristics of the study population

**Figure 2:**
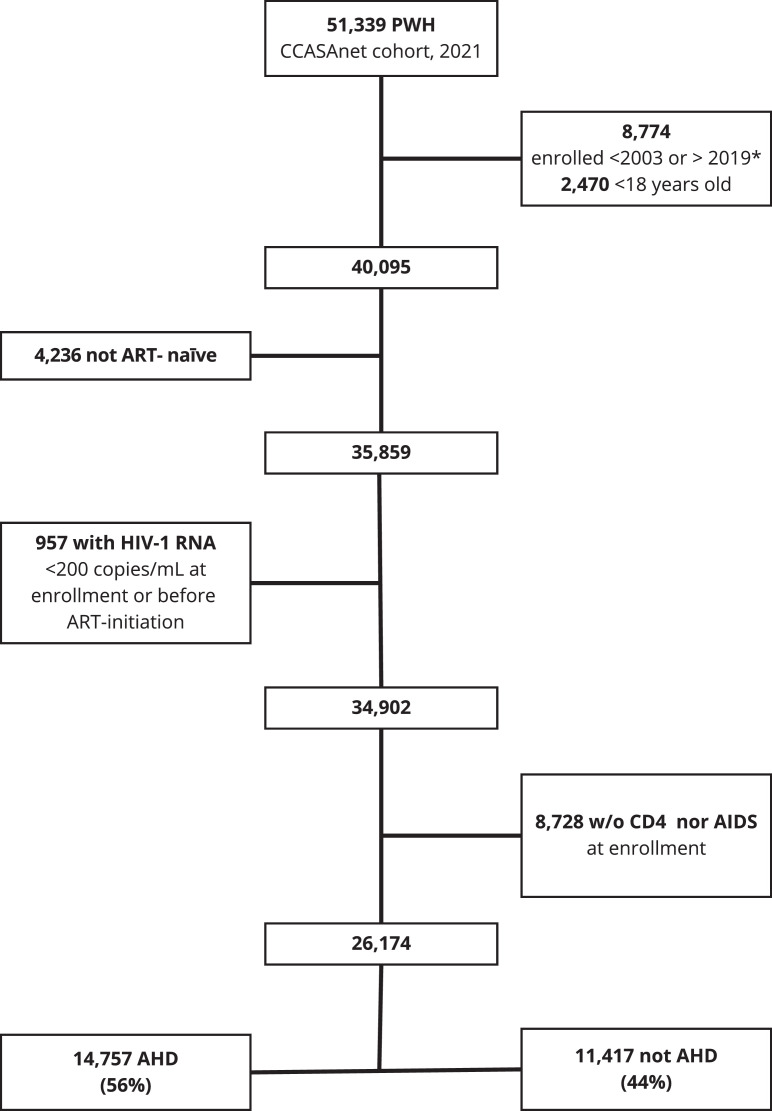
Selection of patients. Note: March 10, 2019, which was 1 year less than the maximum date of enrollment in the site with the lowest period of enrollment in care in the 2021 version of the CCASAnet cohort. AHD, advanced HIV disease; ART, antiretroviral therapy; CCASAnet, Caribbean, Central and South America network for HIV epidemiology; PWH, people with HIV.

The flow diagram of PWH included in the analysis is shown in Figure 2. Of 26,174 PWH included in the study: 14,757 (56%) were enrolled with AHD and 11,417 (44%) without AHD. Of those included, 65% were male at birth, the median age was 34.3 (interquartile range: 27.6-43) years, 13% had less than primary education, and 33% reported heterosexual HIV acquisition risk. People with AHD were older at enrollment (median 36 vs 31 years old), had a lower proportion of patients educated at the university or post-graduate level (17% vs 26%), and had a higher proportion with heterosexual contact as the route of HIV acquisition (38% vs 27%) than those without AHD, respectively.

PWH was followed for a median time of 3.77 years (3.76 years for AHD and 3.79 years for non-AHD), and 93% of PWH started ART (94% among AHD and 91% among non-AHD). A total of 2805 deaths were recorded, 2300 (82%) among PWH with AHD and 505 (18%) among PWH without AHD. We followed 14,497 patients enrolled in care before 2013 and 11,677 patients enrolled in/after 2013, with 61% and 51% of patients in the AHD group being followed during those periods, respectively. Characteristics and outcomes of PWH with and without late presentation by calendar era groups are shown in Table 1. Noticeably, 1705 PWH (6.5% of the cohort) were followed for less than 1 month, of whom 596 were followed for only 1 day. Of all those who followed less than 1 month, 956 (56%) had AHD. In this group with very short follow-up, 675 (71%) were lost to follow-up and 281 died (29%).

### Mean time spent in non-absorbing states

People with AHD who enrolled in care in the first period (before 2013) spent an estimated mean time of 6.21 months in the no-ART stage, 11.44 months in the ART + non-VS stage, and 44.96 months in the ART + VS stage (Table 2). Among PWH without AHD, the mean times in those stages were 14.79 months, 11.06 months, and 51.19 months, respectively. People with AHD enrolled in or after 2013 spent an estimated mean time of 2.68 months in the no-ART stage, 10.93 months in the ART + non-VS stage, and 37.26 months in the ART + VS stage. During this period, the mean times in those stages among people in the non-AHD group were 4.33 months, 11.02 months, and 37.44 months, respectively. Table 2 shows the mean and predicted total time spent in each state by AHD status, stratified by enrollment calendar era.

**Table 2:** Estimated mean time and predicted total time in non-absorbing states by AHD group before and after 2013.

Enrolled before 2013	With AHD	Without AHD
**Total follow-up time in months, mean (95% CI)**	**92.80 (25.9-134)**	**94.03 (47.8-128)**
No-ART, months		
Mean time (95% CI)	6.21 (5.97-6.46)	14.79 (14.22-15.38)
Total time (% of total time)	9.21 (9.9%)	19.77 (21%)
ART + non-VS, months		
Mean time (95% CI)	11.44 (11.05-11.84)	11.06 (10.65-11.48)
Total time (% of total time)	17.23 (18.5%)	15.63 (16.6%)
ART + VS, months		
Mean time (95% CI)	44.96 (43.04-46.95)	51.19 (48.80-53.69)
Total time (% of total time)	43.56 (46.9%)	40.11 (42.6%)

**Enrolled after 2013**		

**Total follow-up time in months, mean (95% CI)**	**22.8 (9.6-44.2)**	**24.5 (10.3-45.9)**
No-ART, months		
Mean time (95% CI)	2.68 (2.52-2.87)	4.33 (4.06-4.63)
Total time (% of total time)	2.94 (12.8%)	4.61 (18.8%)
ART+ non-VS, months		
Mean time (95% CI)	10.93 (10.42-11.47)	11.02 (10.51-11.57)
Total time (% of total time)	7.82 (34%)	7.39 (30%)
ART+ VS, months		
Mean time (95% CI)	37.26 (34.50-40.24)	37.44 (34.72-40.38)
Total time (% of total time)	6.27 (27%)	6.24 (25%)

AHD, advanced HIV disease; CI, confidence interval; ART, antiretroviral therapy; ART + non-VS, on ART without viral suppression (viral load ≥200 copies/ml); ART + VS, on ART with viral suppression (viral load <200 copies/ml); no-ART, enrolled without ART.

Note: We considered non-absorbing states as those that patients can transition out of. Only lost-to-follow-up and death were considered absorbing states, as patients were unable to transition to any other state after entering either of these two states. Mean time in a state is the average time people with HIV spend in a single period of that state conditional on entering the state. Total time in a state is a forecast of the time an individual can spend in the state before transitioning to an absorbing state.

### Probabilities of transitioning at 1 and 5 years of follow-up

**Figure 3:**
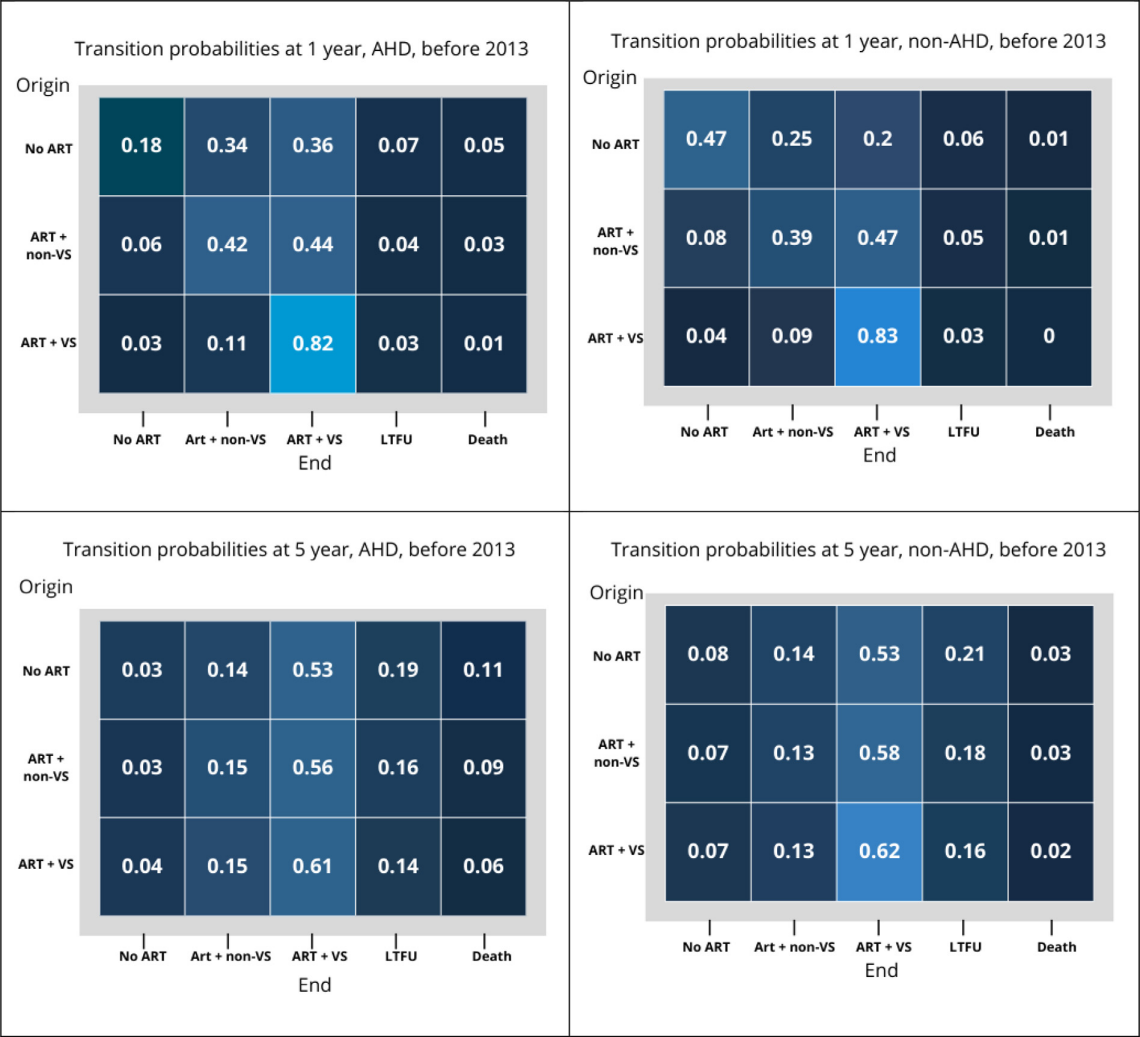

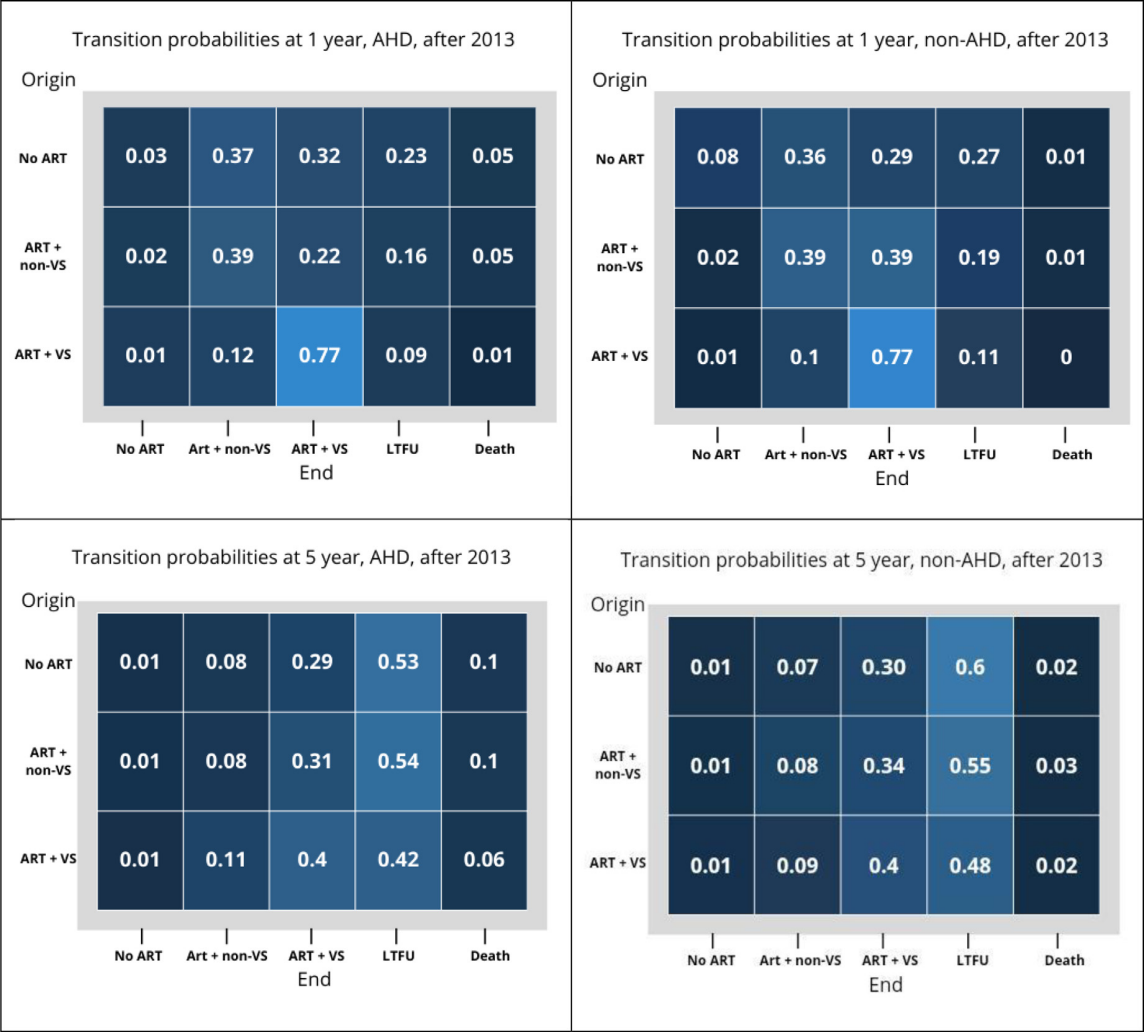
Transition probabilities between state of origin to state of end at 1 and 5 years of follow-up before and after 2013 amond AHD and non-AHD groups. AHD, advanced HIV disease; ART, antiretroviral therapy. Note: 1. No-ART: Enrolled in care not receiving ART; 2. ART + non-VS: on ART without viral suppression (HIV-1 RNA viral load ≥200 copies/ml) or not having an HIV-RNA measurement for more than a year; 3. ART + VS: on ART with viral suppression (ART + VS; viral load <200 copies/ml); 4. LTFU: lost to follow-up (≥1 year between last visit and closure date); or 5. Death.

The probabilities of transitioning across all stages are shown in Figure 3. For example, among those who enrolled in care before 2013 with AHD, the probability of transitioning from no-ART to ART + non-VS after 1 year of follow-up was 0.34. In comparison, among those enrolled without AHD during that period, the probability was 0.25. Similarly, the probabilities of transitioning among those who enrolled before 2013 with AHD from no-ART to the stages of ART + non-VS, ART + VS, LTFU, and death after 5 years of follow-up were 0.14, 0.53, 0.19, and 0.11, respectively. Probabilities of maintaining viral suppression on ART before 2013 were 0.82 at 1 year among AHD, 0.83 among non-AHD, and 0.77 for both groups. These probabilities were even lower at 5 years in both periods: 0.61 for AHD and 0.63 for non-AHD before 2013, and 0.4 in both groups after 2013.

### Adjusted hazard ratios of transitions

We summarized the aHRs of transitioning from and to different stages comparing AHD to non-AHD in the two analyzed calendar periods in Table 3. For example, among those who enrolled in care with AHD before 2013, the aHR associated with transitioning from no-ART to ART + non-VS was 2.09 (95% CI: 2.01-2.16) compared with those enrolled with no-AHD. Overall AHD status was associated with a higher hazard of transitioning from no-ART to all other states among PWH who enrolled before 2013. These aHRs of transitioning to ART + non-VS and ART + VS decreased among those enrolled after 2013, but the aHRs of death significantly increased. AHD status was associated with a higher risk of mortality following all stages in both calendar eras.

**Table 3:** Adjusted hazard ratios for transitioning between states for AHD status vs no AHD at enrollment by calendar period.

		Before 2013	After 2013
From state	To state	aHR (95% CI) (AHD vs no AHD)	aHR (95% CI) (AHD vs no AHD)
No-ART	ART + non-VS	2.09 (2.01-2.16)	1.66 (1.57-1.76)
	ART + VS	3.84 (3.51-4.20)	1.99 (1.72-2.30)
	LTFU	1.73 (1.56-1.92)	1.01 (0.89-1.15)
	Death	8.65 (6.73-11.12)	36.2 (13.22-99.0)
ART + non-VS	No-ART	1.11(1.03-1.19)	1.25 (1.09-1.42)
	ART + VS	0.92 (0.87-0.94)	0.89 (0.85-0.93)
	LTFU	0.90 (0.83-0.97)	1.05 (0.96-1.14)
	Death	2.48 (2.14-2.89)	4.38 (3.44-5.57)
ART + VS	No-ART	1.28 (1.16-1.42)	1.27 (1.07-1.50)
	ART + non-VS	1.16 (1.09-1.21)	1.08 (0.98-1.18)
	LTFU	0.79 (0.73-0.85)	0.75 (0.68-0.84)
	Death	1.79 (1.41-2.26)	1.98 (1.35-2.89)

Note: AHD, advanced HIV disease; aHR, adjusted hazard ratio; ART, antiretroviral therapy; ART + non-VS: on ART without viral suppression (HIV-1 RNA viral load ≥200 copies/ml) or not having an HIV-RNA measurement for more than a year; ART + VS: on ART with viral suppression (ART + VS; viral load <200 copies/ml); Death, HRs were adjusted for sex assigned at birth, age at enrollment (categorized in 3 age groups: <35, 35-50, ≥50 years), and HIV risk acquisition category (heterosexual sexual transmission, same-sex sexual transmission, other, and unknown); LTFU: lost to follow-up (≥1 year between last visit and closure date); No-ART: enrolled in care not receiving ART.

### Sensitivity analyses

After excluding data from Haiti in analyses due to structurally missing VLs, the mean time spent without ART decreased among people without AHD after 2013 and the mean time spent in ART + VS decreased in both groups during this period. The probability of continuing with no-ART at 1 year of follow-up after 2013 slightly increased in both groups, but was the same as in the original analyses after 5 years of follow-up. The probability of transitioning from ART + VS to the lost-to-follow-up stage at 5 years during the period after 2013 decreased, particularly among people with AHD. In addition, the aHR of death from any state, for both periods and comparing AHD to non-AHD also increased.

In additional sensitivity analyses, where we included a “prospective loss” stage, the mean time spent lost before returning to care was 9.79 months among AHD and 12.37 months among non-AHD PWH before 2013 and 8.88 and 9.60 months, respectively, after 2013. The mean time without ART slightly decreased in both periods and groups while time with ART + noVS increased compared to the primary analyses, before 2013 and in both groups. In addition, using an alternative late presentation definition as the primary exposure or adding site as a covariate increased mean times with ART + VS in both groups and periods. This increase was particularly high compared to the primary analysis. Including site was associated with a higher hazard ratio of transitioning between ART + noVS to ART + VS in AHD compared to non-AHD after 2013, in contrast to the results for the primary and other sensitivity analyses (see Supplementary Material).

## Discussion

In this study, we examined the impact that enrolling in care with advanced HIV disease has on achieving critical care continuum benchmarks. We used multi-state multivariable Cox regression models to measure relevant clinical indicators within the framework of the HIV care continuum longitudinally in a cohort of adults living with HIV and receiving care at different sites in Latin America affiliated with CCASAnet. This analytical tool gave us insight into limitations in the provision of care in our region that could be missed by cross-sectional measurements alone. For example, using a cross-sectional approach, we have previously identified that among people enrolled in care at our sites, annual retention improved from 63% to 77% between 2003 and 2012, ART use increased from 74% to 91%, and viral suppression also improved from 54% to 83% during that period [19]. Nonetheless, using this longitudinal approach to assess the continuum of care, we unveiled that for the period before 2013, it took an average of 6 months for PWH with AHD and one-and-a-half years for PWH without AHD to initiate ART after enrollment, and that PWH maintained viral suppression during only about 40% of the observation period (47% in AHD and 42% in non-AHD). Furthermore, we were able to show that in the long term (5 years), the probability of maintaining viral suppression was 61% in people enrolled with AHD and 62% in people enrolled without AHD. This analysis reveals that not only do we need to improve early diagnosis and immediate linkage-to-care and ART initiation but we must also implement strategies to support early retention in care, continued ART adherence, and early identification of events related to ART discontinuation and non-suppressed VLs.

While assessing care continuum indicators using cross-sectional measurements constitutes a helpful method for identifying some key populations who require improved programmatic actions in the treatment as a prevention paradigm [20], the cross-sectional approach censors useful information for people receiving care if they are lost to follow-up or die before the time-point at which estimation occurs [21]. This missing information is relevant, as its exclusion precludes a complete reflection of mortality experiences within the cohort, which comprise a critical clinical indicator. The cross-sectional approach also ignores the status of people not currently receiving care who may contribute disproportionately to ongoing transmission [21]. This is particularly relevant for assessing long-term continuum of care outcomes. Here, we included people followed for less than 1 year (nearly 21% of the total study population, 6.5% of whom were followed for less than 1 month) and analyzed clinically relevant outcomes to improve our understanding of the regional care continuum. These analytical differences explain the lower observed percentages of people in optimal status (on ART with viral suppression) than in previous assessments and show a more realistic appraisal of the situation in our region.

We were also able to identify changes in care continuum stages after the introduction of new ART prescription guidelines and how these differently affected people with AHD and without AHD at enrollment by stratifying the analysis according to the era (before vs at/after these guideline changes occurred). We observed that after the WHO recommendation to initiate ART among people with CD4 <500 cells/µl and prioritize ART initiation in those with more advanced disease in 2013, the mean time without ART was reduced both among AHD and non-AHD, the mean time receiving ART and staying virally suppressed was lengthened in both groups, and the difference between groups observed for this stage was reduced. These positive changes, however, were relevant mainly for PWH without AHD, among whom the time without ART was reduced almost fourfold, on average. Consistent with these results, the hazard of achieving viral suppression on ART among those not previously receiving ART was significantly higher among those with AHD when compared to those without AHD before 2013, but the hazard was reduced after 2013. Worryingly, we also observed worsening long-term indicators of the continuum of care, as the 5-year probability of transitioning to LTFU among virally suppressed participants increased from 16% in people enrolled with AHD and 17% among those without AHD before 2013 to 53% and 59% in the period after 2013, respectively, and persistently high 5-year probabilities of death in almost all stages for people with AHD.

Others have used similar approaches to obtain longitudinal care continuum estimates to more accurately capture the individual, long-term, and heterogeneous interaction between providers and PWH engaged in care [[21], [22], [23]]. Using a multi-state model, Gillis *et al.* [23] estimated the likelihood of transitioning between different states related to clinical care guidelines and provided time spent receiving care in these states. Similarly, Lesko *et al.* [24] added and subtracted cumulative incidence curves to estimate the proportion of patients and average time spent in each stage of the care continuum over time in a cohort of people receiving care in the US and estimated the impact of injection drug use on these parameters. We used multi-state multivariable Cox regression models to approximate the impact that AHD at enrollment had on the continuum of care over the long term, as this is a common condition in our region [[1], [2], [3], [4],^12^], and we suspected that a rapid turnover of this group might have contributed to biased estimates of the continuum of care in cross-sectional analyses.

A limitation of our study is that it does not provide information on the care continuum of individual sites within this heterogeneous group of centers providing HIV care and ART across our region. For instance, we have evidence that in our region and in low- and middle-income countries, VL and CD4 measurements occur less frequently than recommended in the WHO guidelines [25,26]. As we assigned patients without VL measurements for more than 1 year the status of being not virally suppressed, we might have overestimated the overall probabilities of transitioning to the stage of non-viral suppression while receiving ART. Heterogeneity in the size of the patient population at each site might also have led to our estimates being unduly influenced by proportionately larger sites. We tried to address these issues by performing a sensitivity analysis excluding Haiti. Our inferences were robust to changes in laboratory measurement availability, evidenced by similar inferences when excluding the Haitian clinical site (that comprises around half of the participants in the cohort) due to lack of VL measurements during follow-up for most of the study period. We also observed no significant changes in our inferences when introducing a stage for prospective LTFU and when we used the late presentation to care (CD4 <350 cells/µl) instead of AHD as the primary exposure. However, in further sensitivity analyses, including site as a covariate in the regression model, we observed significant increases in time with ART + VS in both exposure groups and periods, and also in the hazard of transitioning from ART + noVS to ART + VS among AHD compared to non-AHD PWH after 2013. This suggests that inter-site heterogeneity may play an important role in our inferences, a topic we must further explore.

We consider this analysis a reasonable initial approach, however, as the information obtained could still be used at each center to improve retention across each continuum stage, and we fully leveraged the rich longitudinal clinical data available within CCASAnet to identify areas of improvement for our programs with lagging progress within the treatment as prevention paradigm: in general, the proportion of time that patients remain virally suppressed while in care is low, and overall, it did not greatly improve over the study period.

In conclusion, using a longitudinal approach to estimate parameters of the care continuum among PWH in Latin America, we identified the fact that, in addition to improving early diagnosis and hastening linkage-to-care and ART initiation, there is a need for strategies to support continued retention in care and ART adherence. This is particularly true in the first year following enrollment in care, and it remains critical to achieving the full potential of treatment as prevention in our region. We also found evidence that suggests that the higher frequency of loss to follow-up and death among people with AHD in our cohort might bias cross-sectional estimates of the continuum of care. This has programmatic implications for measuring the continuum of care in our region.

## Declarations of competing interest

The authors have no competing interests to declare.

## Appendix Supplementary materials

Image, application 1

## Footnotes

Supplementary material associated with this article can be found, in the online version, at doi:10.1016/j.ijregi.2024.100550.
