# Supplementary material for: Transitions through the HIV continuum of care in people enrolling in care with advanced HIV disease in Latin America
Source: IJID Reg. 2024 Dec 18;14:100550. doi: 10.1016/j.ijregi.2024.100550 (PMC11772978; doi:10.1016/j.ijregi.2024.100550)

**Supplementary Material**

**Details of model states definition**

Patients remained “on ART” if they had No-ART interruptions ≥90 days. No-ART state Starts on date of enrollment in care in each site OR the date of registered ART interruption AND ends the date of ART initiation or re-initiation, the date of last visit or the date of death. LTFU and death were considered absorbing states, so once patients reached either of these stages, they were not able to transition to any other state. LTFU starts the date of the last registered visit y this occurred 365 days before date of administrative closure date for each site; and death starts the registered date of death. We used the dates of CD4 and/or VL measurements, medical visits, and ART receipt for each patient to assign their state in the continuum of care at each timepoint. The last measured VLs were carried forward up to one year if a measurement was missing for that period. At time 0, all patients started in either state one (No-ART) or state two (ART+non-VS). ART+non-VS, starts after the date of ART initiation or re-initiation with the first registered HIV-RNA measurement with VL>200 copies/mL and ends the date of the first HIV-RNA measurement <200 copies/mL, the date of last visit or the date of death. ART+VS, starts after the date of ART initiation or re-initiation with the first registered HIV-RNA measurement with VL>200 copies/mL and ends the date of the first HIV-RNA measurement <200 copies/mL, the date of last visit or the date of death. Patients with only one observation available before LTFU or death were included.

**Transition Intensity Matrix**

This matrix shows the proportion of patients transitioning from states 1-3 to states 1-5 since the baseline in the complete cohort and both periods of time.

| **Before 2013** | | | | | |
| --- | --- | --- | --- | --- | --- |
|  | 1.No-ART | 2.ART+non-VS | 3.ART+VS | 4.LTFU | 5.Death |
| 1.No-ART | 0.865561424 | 0.10175750 | 0.01739181 | 0.011640940 | 0.003648324 |
| 2.ART+non-VS | 0.009295964 | 0.94577888 | 0.03411726 | 0.007768007 | 0.0030398872 |
| 3.ART+VS | 0.004324232 | 0.01604828 | 0.97202467 | 0.006646855 | 0.0009559575 |
| 4.LTFU | 0.000000000 | 0.000000000 | 0.000000000 | 0.000000000 | 0.000000000 |
| 5.Death | 0.000000000 | 0.000000000 | 0.000000000 | 0.000000000 | 0.000000000 |
| **After 2013** | | | | | |
| 1.No-ART | 0.624058283 | 0.28090074 | 0.04362944 | 0.04685818 | 0.004553357 |
| 2.ART+non-VS | 0.012628680 | 0.85240312 | 0.09928529 | 0.02885057 | 0.006832339 |
| 3.ART+VS | 0.004542181 | 0.01675226 | 0.96611564 | 0.01153746 | 0.001052457 |
| 4.LTFU | 0.000000000 | 0.000000000 | 0.000000000 | 0.000000000 | 0.000000000 |
| 5.Death | 0.000000000 | 0.000000000 | 0.000000000 | 0.000000000 | 0.000000000 |

**Sensitivity analysis**

1. Excluding Haiti.

When excluding Haitian participants, the estimated mean time spent in the No-ART stage before 2013 was 5.54 months in the AHD group and 18.40 months in the non-AHD group; after 2013, it was 2.74 and 4.77 months, respectively. Estimated mean time spent in the ART+non-VS stage before 2013 was 11.45 months in the AHD and 10.26 in the non-AHD groups; the mean time spent in the ART+VS stage was 45.13 months in the AHD and 51.86 months in the non-AHD groups. Estimates after 2013 were 7.83 months among AHD and 7.18 among non-AHD for the ART+non-VS stage, and 38.30 months among AHD and 40.22 months among non-AHD for the ART+VS stage. (Table S1).

Before 2013, the probability of transitioning from No-ART to ART+non-VS at one year of follow-up in the AHD group was 0.35, to ART+VS was 0.38, to LTFU was 0.07, and to death was 0.05. Before 2013, after five years of follow-up, the probabilities among AHD were 0.14, 0.55, 0.17, and 0.11, respectively. After 2013, probabilities of transitioning from No-ART other stages at one year of follow-up were 0.29, 0.44, 0.18 and 0.04 and 0.29, 0.44, 0.18, and 0.04 at five years of follow-up among AHD (Figure S1).

Before 2013, the aHR for transitioning from No-ART to ART+non-VS for AHD compared to non-AHD was 3.08 (95%CI: 2.93–3.24); from ART+non-VS to ART+VS was 0.86 (95%CI: 0.82–0.89), from ART+VS to LTFU was 0.70 (95%CI: 0.64–0.77). The same transition aHRs after 2013 were 1.81 (95%CI: 1.70–1.93), 0.86 (95%CI: 0.82–0.91), and 0.73 (95%CI: 0.64–0.83). Adjusted hazard ratios for other transitions are shown in Table S2.

**Table S1.** Estimated mean time and predicted total time in non-absorbing states by AHD group before and after 2013 (Excluding Haiti)

| **Enrolled before 2013** | **With Advanced HIV Disease** | **Without Advanced HIV Disease** |
| --- | --- | --- |
| Total follow-up time in months, mean (95%CI) | 105.7 (47.97 – 145.13) | 103.7 (67.37 – 139.22) |
| No-ART, months  Mean time (95%CI)  Total time (% of total time) | 5.54 (5.29 – 5.80)  8.74 (8.3%) | 18.40 (17.49 – 19.35)  25.14 (24.2%) |
| ART+ non-VS, months  Mean time (95%CI)  Total time (% of total time) | 11.45 (11.01 – 11.91)  19.27 (18%) | 10.26 (9.84 – 10.69)  14.53 (14%) |
| ART+ VS, months  Mean time (95%CI)  Total time (% of total time) | 45.32 (43.26 – 47.40)  51.38 (48%) | 51.86 (49.24 – 54.62)  43.05 (41.5%) |
| **Enrolled after 2013** |  |  |
| Total follow-up time in months, mean (95%CI) | 29.13 (12.43 – 51.77) | 23.37 (11.63 – 44.59) |
| No-ART, months  Mean time (95%CI)  Total time (% of total time) | 2.75 (2.55 – 2.95)  3.28 (11.2%) | 4.77 (4.43 – 5.15)  5.26 (22%) |
| ART+ non-VS, months  Mean time (95%CI)  Total time (% of total time) | 7.83 (7.32 – 8.37)  7.32 (25%) | 7.18 (6.73 – 7.67)  5.35 (23%) |
| ART+ VS, months  Mean time (95%CI)  Total time (% of total time) | 38.30 (34.72 – 42.27)  11.78 (40%) | 40.22 (36.54 – 44.26)  7.67 (33%) |

***Note****:* If a patient can exit one state and enter another, the initial state is a non-absorbing state; on the other hand, a state which is not possible to exit, such as LTFU or Death, is an absorbing state. 1. No-ART: Enrolled in care not receiving ART; 2. ART + non-VS: on ART without viral suppression (HIV-1 RNA viral load [VL]≥200 copies/mL) or not having an HIV-RNA measurement for more than a year; 3. ART+ VS: on ART with viral suppression (ART+VS; VL<200 copies/mL); 4. LTFU: lost to follow-up (≥1 year between last visit and closure date); or 5. Death.

**Table S2.** Adjusted hazard ratios for transitioning between states for AHD status vs. no AHD at enrollment by calendar period (Excluding Haiti)

|  |  | **Before 2013** | **After 2013** |
| --- | --- | --- | --- |
| **From State** | **To State** | **aHR (95% CI)**  **(AHD vs. no AHD)** | **aHR (95% CI)**  **(AHD vs. no AHD)** |
| No-ART | ART+non-VS | 3.08 (2.93 – 3.24) | 1.81 (1.70 – 1.93) |
|  | ART+VS | 4.61 (4.17 – 5.08) | 2.02 (1.75 – 2.35) |
|  | LTFU | 1.94 (1.68 – 2.24) | 0.99 (0.87 – 1.14) |
|  | Death | 10.73 (7.90 – 14.58) | 40.34 (14.91 – 109.11) |
| ART+non-VS | No-ART | 0.95 (0.86 – 0.89) | 1.07 (0.93 – 1.24) |
|  | ART+VS | 0.86 (0.82 – 0.89) | 0.86 (0.82 – 0.91) |
|  | LTFU | 0.76 (0.64 – 0.90) | 0.86 (0.74 – 0.99) |
|  | Death | 3.04 (2.32 – 4.00) | 8.81 (5.25 – 14.77) |
| ART+VS | No-ART | 1.27 (1.14 – 1.40) | 1.28 (1.08 – 1.53) |
|  | ART+non-VS | 1.18 (1.12 – 1.25) | 1.17 (1.05 – 1.29) |
|  | LTFU | 0.70 (0.64 – 0.77) | 0.73 (0.64 – 0.83) |
|  | Death | 2.23 (1.68 – 2.98) | 2.55 (1.54 – 4.22) |

***Note****:* 1. No-ART: Enrolled in care not receiving ART; 2. ART + non-VS: on ART without viral suppression (HIV-1 RNA viral load [VL]≥200 copies/mL) or not having an HIV-RNA measurement for more than a year; 3. ART+ VS: on ART with viral suppression (ART+VS; VL<200 copies/mL); 4. LTFU: lost to follow-up (≥1 year between last visit and closure date); or 5. Death. Hazard Ratios were adjusted for sex assigned at birth, age at enrollment (categorized in 3 age groups: <35, 35-50, ≥50 years), and HIV risk acquisition category (heterosexual sexual transmission, same-sex sexual transmission, other, and unknown).

**Figure S1. Transition probabilities between state of origin to state of end at 1 and 5 years of follow–up before and after 2013 among AHD and non-AHD groups excluding Haiti.**

| **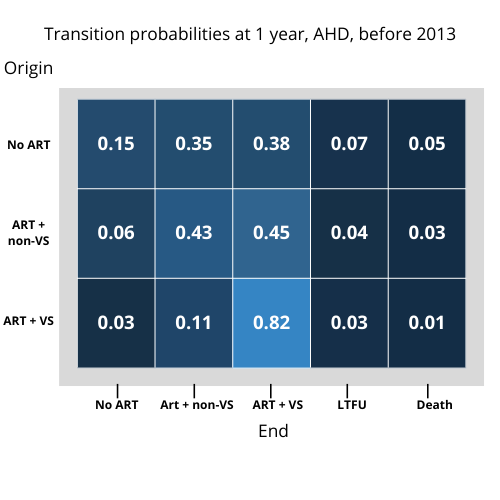** | **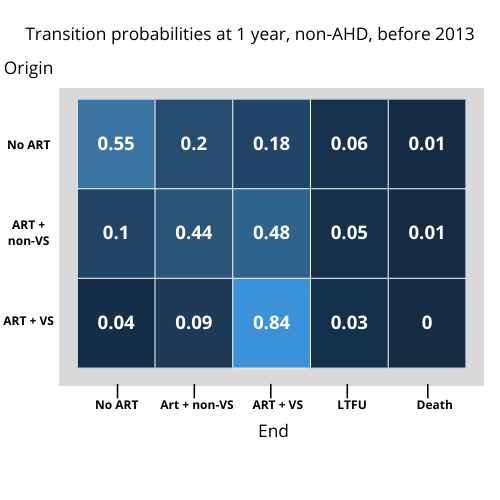** |
| --- | --- |
| **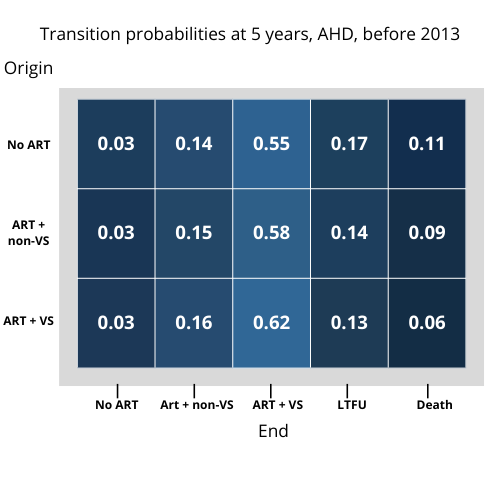** | **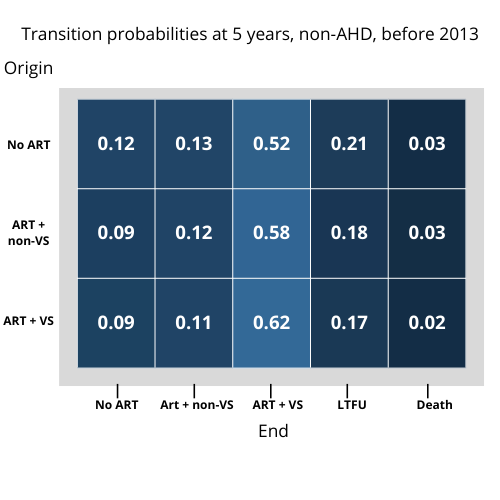** |
| 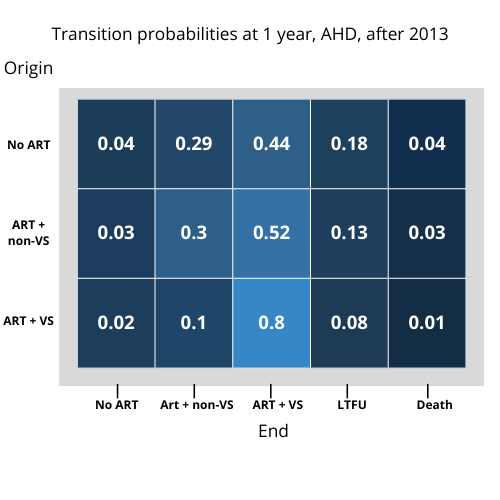 | **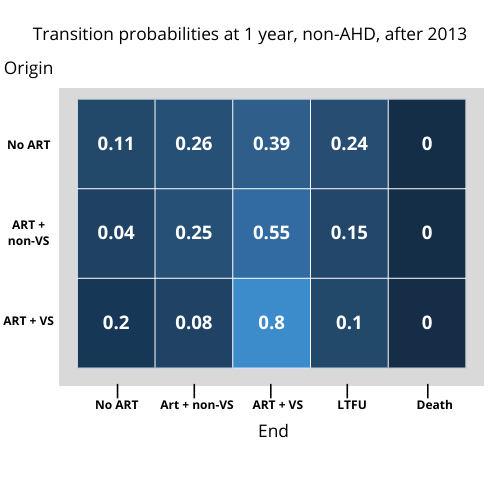** |
| 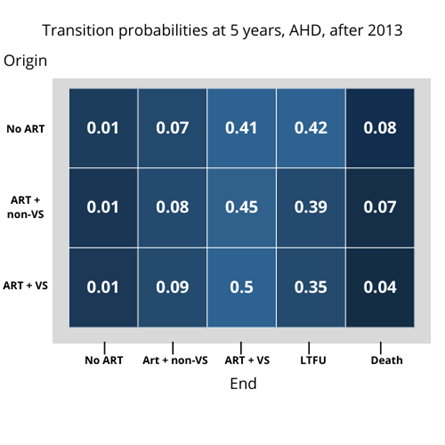 | **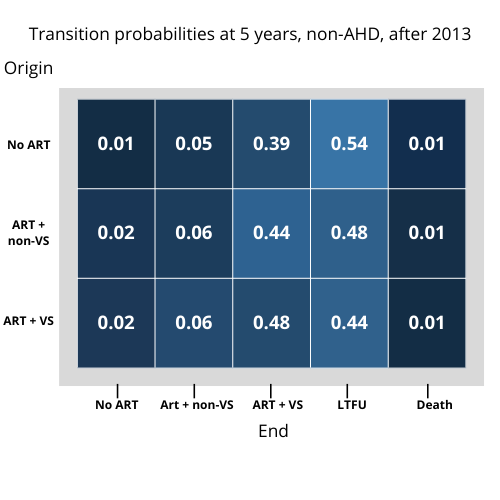** |

***Note:*** 1. No-ART: Enrolled in care not receiving ART; 2. ART + non-VS: on ART without viral suppression (HIV-1 RNA viral load [VL]≥200 copies/mL) or not having an HIV-RNA measurement for more than a year; 3. ART+ VS: on ART with viral suppression (ART+VS; VL<200 copies/mL); 4. LTFU: lost to follow-up (≥1 year between last visit and closure date); or 5. Death

**Factors associated to hazards of transitioning***.*

Adjusted hazard ratios for transitioning between states for AHD, sex, age, and HIV risk acquisition covariates, for the complete study population, are shown in Table S3. Being older than 50 years old compared to younger than 35 was associated with a higher adjusted hazard of transitioning from No-ART to ART+VS, before 2013: aHR=1.37 (95%CI: 1.29–1.46) but not after 2013 aHR=1.08 (95%CI: 0.98–1.19), respectively. Older age was also associated with a higher adjusted hazard of transitioning to ART+VS from ART+non-VS, both before and after 2013, for people older than 50 vs younger than 35: aHR=1.37 (95%CI: 1.29–1.46), and; aHR=1.13 (95%CI: 1.04–1.22) respectively. Having MSM compared to heterosexual HIV risk of acquisition was associated with a higher adjusted hazard of transitioning from ART+non-VS to ART+VS, both before and after 2013: aHR=1.15 (95%CI: 1.09–1.20) and aHR=1.76 (95%CI: 1.66 – 1.88); having MSM compared to heterosexual HIV acquisition risk was also associated with an increased adjusted hazard of transitioning from ART+VS to LTFU before 2013 : aHR=2.11 (95%CI: 1.88 – 2.37) .(Table S3)

1. Including a new prospective loss state.

We add a state to describe the frequency of longer periods where people seem not being in clinical care. We called it: *Prospective Loss* state, and we defined it starts once a period longer than 365 days was recorded, with a next registered visit after for any reason or death. The figure S2 shows the model with the possible transitions including this new state.

The estimated mean time spent in the No-ART stage before 2013 was 5.31 months in the AHD group and 11.69 months in the non-AHD group; after 2013, it was 2.27 and 3.59 months, respectively. Estimated mean time spent in the ART+non-VS stage before 2013 was 19.94 months in the AHD and 20.71 in the non-AHD groups; the mean time spent in the ART+VS stage was 46.23 months in the AHD and 46.31 months in the non-AHD groups, after 2013. The mean time for Prospective Loss was 9.79 months in the AHD and 12.37 months in the non-AHD, before 2013. Estimates after 2013 were 9.08 months among AHD and 8.63 among non-AHD for the ART+non-VS stage, and 39.11 months among AHD and 38.45 months among non-AHD for the ART+VS stage. In Prospective Loss state, were 8.88 ,months in AHD and 9.60 in non-AHD (Table S4).

Before 2013, the aHR for transitioning from No-ART to ART+non-VS for AHD compared to non-AHD was 2.19 (95%CI: 2.11–2.27); from ART+non-VS to ART+VS was 1.07 (95%CI: 1.03–1.11), from ART+VS to Prospective Loss was 0.79 (95%CI:0.73- 0.85) and from Prospective Loss to LTFU was 1.68 (95%CI: 1.45–1.93). The same transition aHRs after 2013 were 1.72 (95%CI: 1.63–1.82), 0.84 (95%CI: 0.80-0.88), 0.92 (95%CI:0.81 -1.04) and 0.97 (95%CI: 0.79–1.19). Adjusted hazard ratios for other transitions are shown in Table S5.

1. Using LP definition of CD4<350 and/or AIDS.

Instead of AHD we used the late presentation definition of having CD4 count lower than 350 and/or AIDS at enrollment; and it was the only covariate we included in the model to estimate the continuum. The estimated mean time spent in the No-ART stage before 2013 was 6.53 months in the LP group and 20.34 months in the non-LP group; after 2013, it was 2.54 and 5.11 months, respectively. Estimated mean time spent in the ART+non-VS stage before 2013 was 24.39 months in the LP and 21.71 in the non-LP groups; the mean time spent in the ART+VS stage was 51.62 months in the LP and 53.72 months in the non-LP groups. Estimates after 2013 were 11.22 months among LP and 10.63 among non-LP for the ART+non-VS stage, and 43.30 months among LP and 43.56 months among non-LP for the ART+VS stage. (Table S4).

Before 2013, the aHR for transitioning from No-ART to ART+non-VS for LP compared to non-LP was 3.06 (95%CI: 2.93–3.19); from ART+non-VS to ART+VS was 0.81 (95%CI: 0.78–0.85) and from ART+VS to Loss to LTFU was 0.81 (95%CI: 0.74–0.88). The same transition aHRs after 2013 were 2.18 (95%CI: 2.05–2.32), 0.84 (95%CI: 0.80-0.89), and 0.87 (95%CI: 0.79–0.98). Adjusted hazard ratios for other transitions are shown in Table S5.

1. Including site as a covariate in the original model.

In this model with the original five states and the definition of AHD, we added the covariate site to the list of AHD, sex, age and mode of transmission. The estimated mean time spent in the No-ART stage before 2013 was 6.89 months in the AHD group and 15.26 months in the non-AHD group; after 2013, it was 2.78 and 4.44 months, respectively. Estimated mean time spent in the ART+non-VS stage before 2013 was 9.38 months in the AHD and 8.72 in the non-AHD groups; the mean time spent in the ART+VS stage was 84.25 months in the AHD and 86.99 months in the non-AHD groups. Estimates after 2013 were 3.81 months among AHD and 3.56 among non-AHD for the ART+non-VS stage, and 178.79 months among AHD and 170.45 months among non-AHD for the ART+VS stage. (Table S4).

Before 2013, the aHR for transitioning from No-ART to ART+non-VS for AHD compared to non-AHD was 2.10 (95%CI: 2.02–2.18); from ART+non-VS to ART+VS was 0.90 (95%CI: 0.87–0.94) and from ART+VS to Loss to LTFU was 0.87 (95%CI: 0.79–0.96). The same transition aHRs after 2013 were 0.88 (95%CI: 0.81–0.95), 1.12 (95%CI: 1.05-1.20), and 0.87 (95%CI: 0.75–1.00). Adjusted hazard ratios for other transitions are shown in Table S5.

We compared the mean time in non-absorbing states estimated in each sensitivity analyses with the original analyses, and the changes in the hazard ratio for AHD compared with non-AHD in some transitions in the figures S3 and S4.

**Figure S2.** Model structure including new Prospective Loss state.

**Linkage**

Note : 1. No-ART: in care but not receiving ART; 2. ART+non-VS: on ART without viral suppression (HIV-1 RNA viral load [VL]≥200 copies/mL) or on ART but not having a VL for more than a year); 3. ART+VS: on ART with viral suppression (VL<200 copies/mL); 4. Prosp. Loss: Prospective Loss: starts once a period longer than 365 days was recorded, with a next registered visit after for any reason or death 5. LTFU: loss to follow-up (≥1 year between last visit and cohort closure date); or 6. All-cause death .

**Table S3. Adjusted hazard ratios for transitioning between states for sex, age, and HIV risk acquisition covariates.**

|  |  | **Male vs Female** | | **Age >50 vs Age <35** | | **Mode MSM vs Heterosexual** | |
| --- | --- | --- | --- | --- | --- | --- | --- |
|  |  | **Before 2013** | **After 2013** | **Before 2013** | **After 2013** | **Before 2013** | **After 2013** |
| **From State** | **To State** | **aHR (95%CI)** | **aHR (95%CI)** | **aHR (95%CI)** | **aHR (95%CI)** | **aHR (95%CI)** | **aHR (95%CI)** |
| No-ART | ART+non-VS | 1.01  (0.97 – 1.05) | 0.88  (0.81 – 0.95) | 1.37  (1.29 – 1.46) | 1.08  (0.98 – 1.19) | 0.88  (0.83 – 0.93) | 0.97  (0.90 – 1.04) |
|  | ART+VS | 1.01  (0.91 – 1.11) | 1.09  (0.88 – 1.35) | 1.41  (1.22 – 1.63) | 1.17  (0.91 – 1.51) | 0.89  (0.80 – 0.99) | 1.03  (0.86 – 1.22) |
|  | LTFU | 1.12  (1.00 – 1.26) | 0.99  (0.82 – 1.20) | 1.09  (0.92 – 1.30) | 1.13  (0.91 – 1.40) | 1.04  (0.88 – 1.23) | 1.42  (1.21 – 1.67) |
|  | Death | 1.22  (0.99 – 1.49) | 1.54  (0.94 – 2.51) | 2.04  (1.57 – 2.65) | 2.08  (1.21 – 3.58) | 0.87  (0.69 – 1.17) | 0.65  (0.40 – 1.04) |
| ART+non-VS | No-ART | 1.05  (0.97 – 1.28) | 1.19  (1.00 – 1.41) | 0.81  (0.75 – 0.89) | 0.75  (0.59 – 0.95) | 0.98  (0.88 – 1.08) | 1.58  (1.34 – 1.87) |
|  | ART+VS | 1.13  (1.09 – 1.18) | 1.12  (1.05 – 1.20) | 1.37  (1.29 – 1.46) | 1.13  (1.04 – 1.22) | 1.15  (1.10 – 1.20) | 1.76  (1.66 – 1.88) |
|  | LTFU | 1.04  (0.97 – 1.13) | 0.77  (0.69 – 0.85) | 0.86  (0.76 – 0.97) | 0.95  (0.82 – 1.09) | 1.03  (0.85 – 1.24) | 1.05  (0.92 – 1.19) |
|  | Death | 1.29  (1.13 – 1.46) | 1.25  (1.04 – 1.52) | 1.74  (1.45 – 2.06) | 2.52  (2.01 – 3.16) | 0.81  (0.65 – 0.99) | 0.59  (0.45 – 0.79) |
| ART+VS | No-ART | 0.83  (0.79 – 0.87) | 1.10  (0.87 – 1.39) | 0.60  (0.51 – 0.72) | 0.72  (0.54 – 0.97) | 0.69  (0.61 – 0.77) | 0.82  (0.66 – 1.00) |
|  | ART+non-VS | 0.89  (0.80 – 1.00) | 0.93  (0.84 – 1.04) | 0.68  (0.62 – 0.74) | 0.80  (0.69 – 0.93) | 0.77  (0.72 – 0.82) | 0.69  (0.62 – 0.78) |
|  | LTFU | 0.87  (0.79 – 0.96) | 0.87  (0.75 – 1.00) | 0.97  (0.86 – 1.09) | 1.14  (0.96 – 1.35) | 2.11  (1.88 – 2.37) | 1.14  (0.99 – 1.31) |
|  | Death | 1.01  (0.86 – 1.38) | 1.30  (0.86 – 1.98) | 3.99  (3.05 – 5.22) | 4.72  (2.96 – 7.51) | 1.02  (0.79 – 1.32) | 0.62  (0.39 – 0.97) |

***Note:*** 1. No-ART: Enrolled in care not receiving ART; 2. ART + non-VS: on ART without viral suppression (HIV-1 RNA viral load [VL]≥200 copies/mL) or not having an HIV-RNA measurement for more than a year; 3. ART+ VS: on ART with viral suppression (ART+VS; VL<200 copies/mL); 4. LTFU: lost to follow-up (≥1 year between last visit and closure date); or 5. Death.

**Table S4.** Estimated mean time and predicted total time in non-absorbing states by AHD group before and after 2013 in different sensitivity analyses

|  | **Including a Prospective Loss state** | | **Using definition of CD4<350 and/or AIDS** | | **Including site as covariate** | |
| --- | --- | --- | --- | --- | --- | --- |
| **Enrolled before 2013** | **With AHD** | **Without AHD** | **With LP** | **Without LP** | **With AHD** | **Without AHD** |
| No-ART, months  Mean time (95%CI) | 5.31  (5.21 – 5.41) | 11.69  (11.42 – 11.96) | 6.53  (6.42 – 6.64) | 20.34  (19.68 – 21.03) | 6.89  (6.07 – 7.79) | 15.26  (13.57 – 17.17) |
| ART+ non-VS, months  Mean time (95%CI) | 19.94  (19.62 – 20.28) | 20.71  (20.26 – 21.17) | 24.39  (24.02 – 24.76) | 21.71  (21.03 – 22.42) | 9.38  (8.54 – 10.30) | 8.72  (7.97 – 9.55) |
| ART+ VS, months  Mean time (95%CI) | 46.23  (45.25 – 47.22) | 46.31  (44.99 – 47.66) | 51.62  (50.59 – 52.65) | 53.72  (51.51 – 56.02) | 84.25  (74.66 – 95.1) | 86.99  (77.34 – 97.83) |
| Prospective Loss,  Months  Mean time (95%CI) | 9.79  (9.46 – 10.12) | 12.37  (11.94 – 12.82) |  |  |  |  |
| **Enrolled after 2013** | **With AHD** | **Without AHD** | **With LP** | **Without LP** | **With AHD** | **Without AHD** |
| No-ART, months  Mean time (95%CI) | 2.27  (2.19 – 2.35) | 3.59  (3.47 – 3.71) | 2.54  (2.47 – 2.61) | 5.11  (4.90 – 5.33) | 2.78  (2.23 – 3.46) | 4.44  (3.59 – 5.48) |
| ART+ non-VS, months  Mean time (95%CI) | 9.08  (8.85 – 9.32) | 8.63  (8.41 – 8.87) | 11.22  (10.96 – 11.48) | 10.63  (10.27 – 11.00) | 3.81  (3.21 – 4.52) | 3.56  (3.01 – 4.22) |
| ART+ VS, months  Mean time (95%CI) | 39.11  (37.55 – 40.73) | 38.45  (36.94 – 40.03) | 43.30  (41.77 – 44.89) | 43.56  (41.28 – 45.96) | 178.79  (127.7 – 250.4) | 170.45  (122.8 – 236.5) |
| Prospective Loss,  Months  Mean time (95%CI) | 8.88  (8.38 – 9.41) | 9.60  (9.09 – 10.14) |  |  |  |  |

***Note:*** 1. No-ART: Enrolled in care not receiving ART; 2. ART + non-VS: on ART without viral suppression (HIV-1 RNA viral load [VL]≥200 copies/mL) or not having an HIV-RNA measurement for more than a year; 3. ART+ VS: on ART with viral suppression (ART+VS; VL<200 copies/mL); 4. Prosp. Loss: Prospective Loss: starts once a period longer than 365 days was recorded, with a next registered visit after for any reason or death 5. LTFU: loss to follow-up (≥1 year between last visit and cohort closure date); or 6. All-cause death .

**Table S5. Adjusted hazard ratios for transitioning between states for AHD status and different sensitivity analyses.**

|  |  | **Including a Prospective Loss State** | | **Using LP definition of CD4<350 and/or AIDS** | | **Including site as covariate** | |
| --- | --- | --- | --- | --- | --- | --- | --- |
|  |  | **Before 2013** | **After 2013** | **Before 2013** | **After 2013** | **Before 2013** | **After 2013** |
| **From State** | **To State** | **aHR (95%CI)** | **aHR (95%CI)** | **aHR (95%CI)** | **aHR (95%CI)** | **aHR (95%CI)** | **aHR (95%CI)** |
| No-ART | ART+non-VS | 2.19  (2.11 – 2.27) | 1.72  (1.63 – 1.82) | 3.06  (2.93 – 3.19) | 2.18  (2.05 – 2.32) | 2.10  (2.02 – 2.18) | 0.88  (0.81 – 0.95) |
|  | ART+VS | 3.23  (2.96 – 3.53) | 1.92  (1.67 – 2.22) | 3.59  (3.24 – 3.99) | 2.32  (1.99 – 2.70) | 3.84  (3.48 – 4.22) | 1.09  (0.88 – 1.35) |
|  | Prospective  Loss | 0.88  (0.78 – 0.99) | 0.67  (0.51 – 0.88) |  |  |  |  |
|  | LTFU | 1.92  (1.73 – 2.12) | 0.92  (0.81 – 1.04) | 2.38  (2.13 – 2.66) | 1.19  (1.06 – 1.35) | 1.52  (1.37 – 1.69) | 0.99  (0.82 – 1.20) |
|  | Death | 9.56  (7.33 – 12.47) | 40.77  (15.12 – 109.9) | 8.35  (6.13 – 11.38) | 45.13  (11.99 – 169.83) | 8.98  (6.92 – 11.64) | 1.54  (0.94 – 2.51) |
| ART+non-VS | No-ART | 1.16  (1.08 – 1.25) | 1.14  (0.99 – 1.30) | 1.02  (0.94 – 1.12) | 1.18  (1.02 – 1.36) | 1.16  (1.08 – 1.25) | 1.19  (1.00 – 1.41) |
|  | ART+VS | 1.07  (1.03 – 1.11) | 0.84  (0.80 – 0.88) | 0.81  (0.78 – 0.85) | 0.84  (0.80 – 0.89) | 0.90  (0.87 – 0.94) | 1.12  (1.05 – 1.20) |
|  | Prospective  Loss | 0.74  (0.69 – 0.80) | 0.91  (0.81 – 1.01) |  |  |  |  |
|  | LTFU | 0.81  (0.75 – 0.87) | 1.00  (0.92 – 1.09) | 0.85  (0.78 – 0.93) | 0.98  (0.89 – 1.07) | 0.89  (0.82 – 0.96) | 0.77  (0.69 – 0.85) |
|  | Death | 2.83  (2.41 – 3.32) | 5.79  (4.41 – 7.62) | 2.44  (1.96 – 3.04) | 4.44  (3.26 – 6.05) | 2.46  (2.11 – 2.86) | 1.25  (1.04 – 1.52) |
| ART+VS | No-ART | 1.30  (1.17 – 1.45) | 1.25  (1.05 – 1.48) | 1.00  (0.89 – 1.13) | 1.02  (0.85 – 1.21) | 1.22  (1.10 – 1.36) | 1.10  (0.87 – 1.39) |
|  | ART+non-VS | 1.19  (1.13 – 1.26) | 1.14  (1.04 – 1.25) | 1.15  (1.07 – 1.22) | 1.05  (0.96 – 1.16) | 1.09  (1.03 – 1.15) | 0.93  (0.84 – 1.04) |
|  | Prospective  Loss | 0.79  (0.73 – 0.85) | 0.92  (0.81 – 1.04) |  |  |  |  |
|  | LTFU | 0.68  (0.63 – 0.74) | 0.72  (0.64 – 0.79) | 0.81  (0.74 – 0.88) | 0.87  (0.79 – 0.98) | 0.87  (0.79 – 0.96) | 0.87  (0.75 – 1.00) |
|  | Death | 2.10  (1.63 – 2.72) | 2.49  (1.67 – 3.70) | 1.79  (1.32 – 2.42) | 2.31  (1.46 – 3.64) | 1.83  (1.44 – 2.33) | 1.30  (0.86 – 1.98) |
| Prospective  Loss | No-ART | 0.80  (0.71 – 0.89) | 0.68  (0.52 – 0.89) |  |  |  |  |
|  | ART+non-VS | 1.33  (1.23 – 1.44) | 1.18  (1.05 – 1.32) |  |  |  |  |
|  | ART+VS | 1.35  (1.22 – 1.49) | 0.99  (0.84 – 1.17) |  |  |  |  |
|  | LTFU | 1.68  (1.45 – 1.93) | 0.97  (0.79 – 1.19) |  |  |  |  |
|  | Death | 2.25  (1.66 – 3.06) | 2.98  (1.90 – 4.68) |  |  |  |  |

***Note:*** 1. No-ART: Enrolled in care not receiving ART; 2. ART + non-VS: on ART without viral suppression (HIV-1 RNA viral load [VL]≥200 copies/mL) or not having an HIV-RNA measurement for more than a year; 3. ART+ VS: on ART with viral suppression (ART+VS; VL<200 copies/mL); 4. Prosp. Loss: Prospective Loss: starts once a period longer than 365 days was recorded, with a next registered visit after for any reason or death 5. LTFU: loss to follow-up (≥1 year between last visit and cohort closure date); or 6. All-cause death .

**Figure S3. Mean Times by non-absorbing state, period and group of AHD (LP) in different sensitivity analyses.**

| 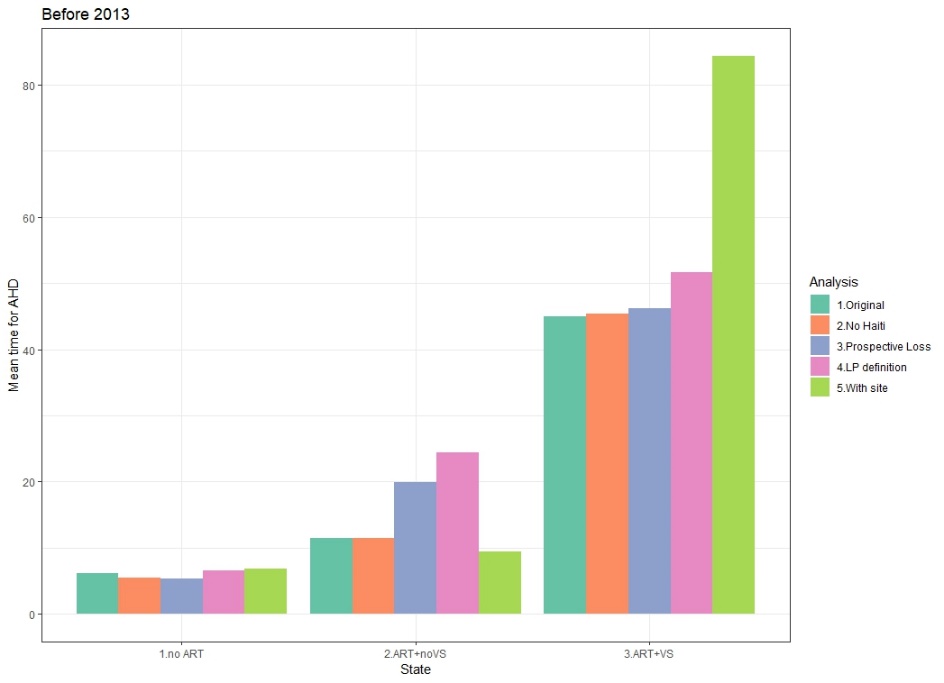 | 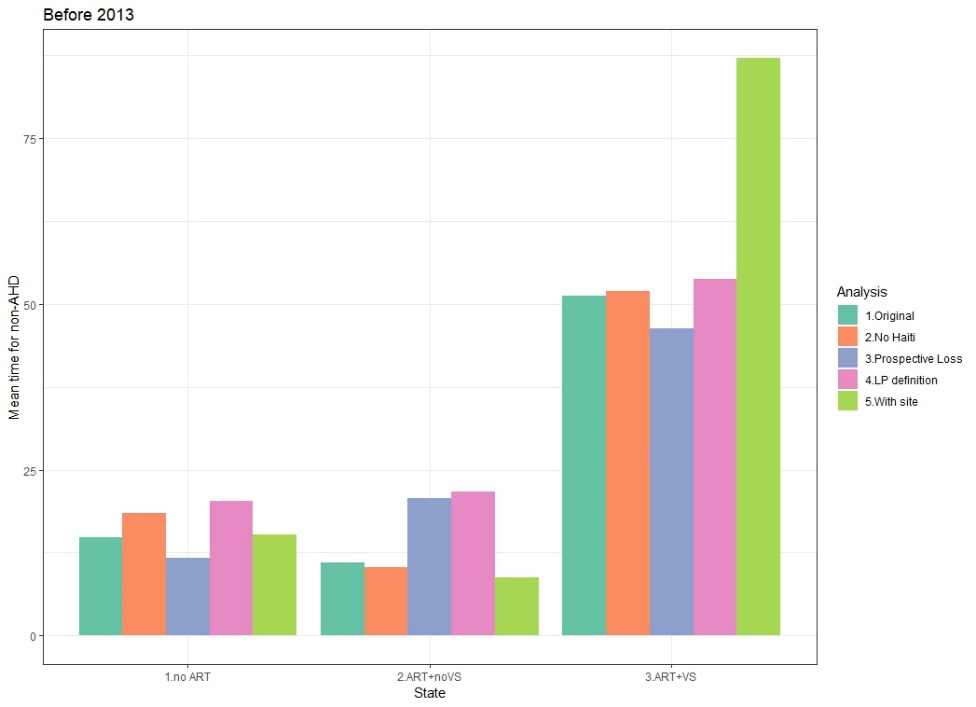 |
| --- | --- |
| 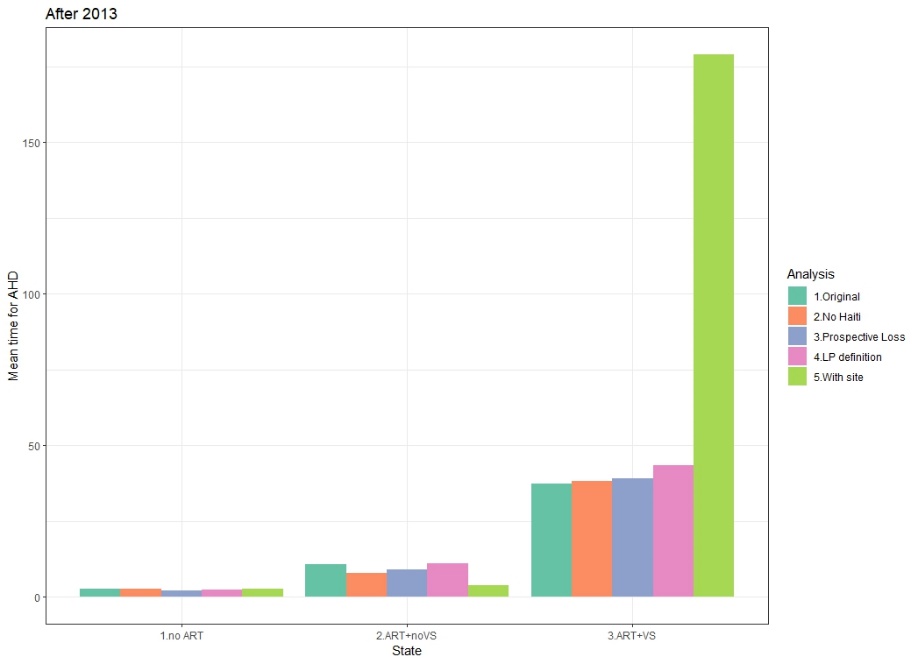 | 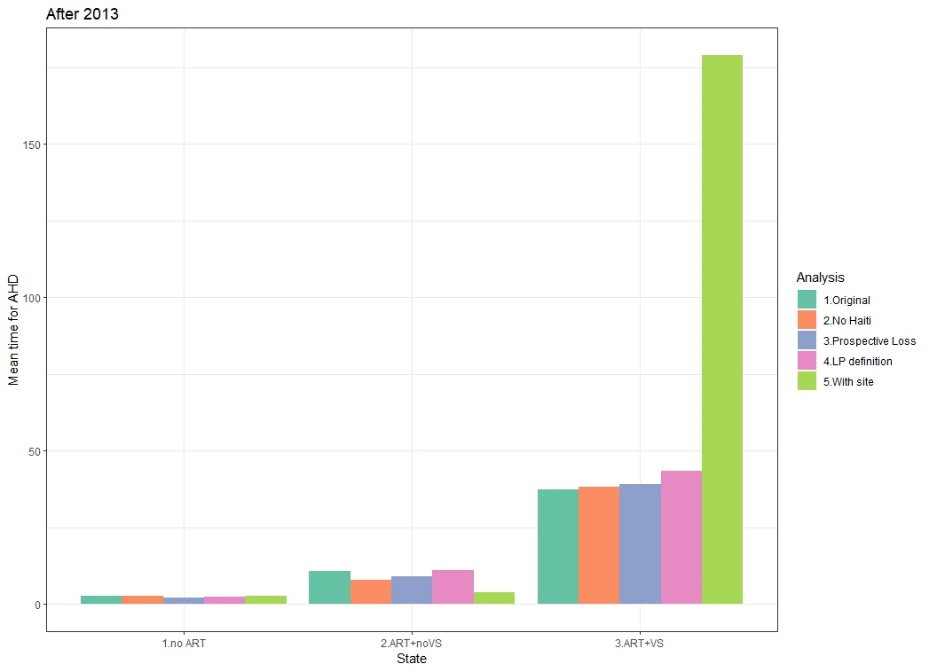 |

**Figure S4. Hazard ratios for transitioning between stages for AHD vs non-AHD groups by sensitivity analysis and periods of study.**


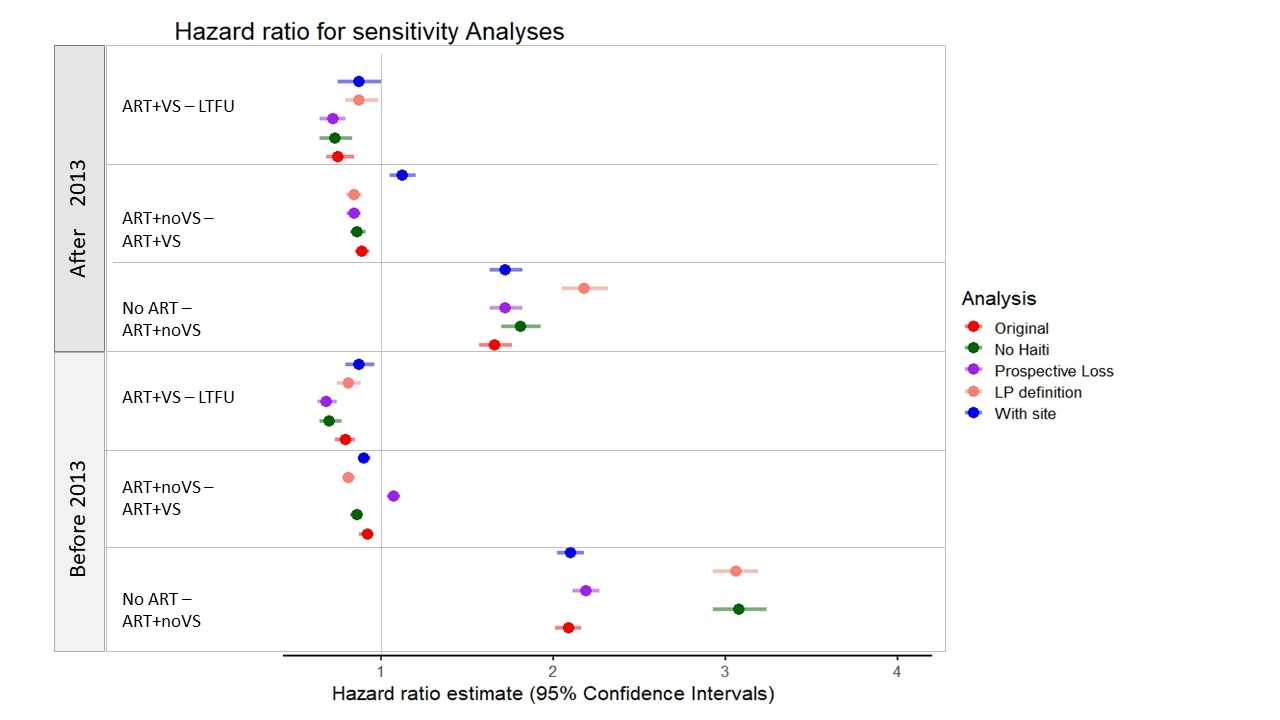

Supplement: Supplementary file 1 [file mmc1.docx]
